# Prevention and control of hospital-acquired infections with multidrug-resistant organism: A review

**DOI:** 10.1097/MD.0000000000037018

**Published:** 2024-01-26

**Authors:** Binghui Ji, Weijiang Ye

**Affiliations:** a Third Clinical Medical College of Zhejiang Chinese Medical University, Hangzhou, China; b The Rehabilitation Hospital of the Third Affiliated Hospital of Zhejiang Chinese Medical University, Hangzhou, China.

**Keywords:** acquired infections, hospital, multidrug, prevention, resistant organism

## Abstract

Multidrug-resistant is defined as nonsusceptibility to at least 1 agent in 3 or more antimicrobial categories. Controlling the spread of drug-resistant organisms is a key step in the management of hospital-acquired infections (HAIs). To review the progress of research on the prevention and control of HAIs with multidrug-resistant organism (MDRO) in the past 5 years, and to provide reference for the development of comprehensive measures for the prevention and control of HAIs with MDRO. We conducted a search in the PUBMED database for studies related to MDRO and HAIs from 2018 to 2023, then integrated this data with information sourced from the U.S.A. The Centers for Disease Control and Prevention. Utilizing information technology to monitor and provide feedback on hand hygiene practices can enhance compliance. Environmental disinfection techniques such as ultraviolet or hydrogen peroxide demonstrate potential in reducing MDRO transmission. While some studies support that contact isolation measures for MDRO-infected or colonized patients can reduce HAIs, others do not confirm this outcome. Approaches for MDRO colonization among patients or physicians may mitigate MDRO transmission risk. Implementing clusterization interventions proves to enhance efficiency and cost-effectiveness in preventing and controlling MDRO. Early screening for pathogen species emerges as a valuable strategy aiding in antimicrobial use control. Combined with evidence from the literature, implementing clusterization interventions that include measures such as monitoring and feedback on hand hygiene and improved environmental disinfection techniques can help prevent and control HAIs with MDRO. However, further clinical studies are needed to validate the optimal clusterization intervention.

## 1. Introduction

Multidrug-resistant (MDR) is defined as nonsusceptibility to at least 1 agent in 3 or more antimicrobial categories.^[1]^ The 2019 U.S. Antimicrobial Resistance Threat Report^[2]^ reveals that over 2.8 million infections in the United States of America are triggered by drug-resistant bacteria per annum, leading to over 35,000 deaths. In the European Union, drug-resistant bacteria cause more than 670,000 infections and approximately 33,000 deaths per year.^[3]^ During the corona virus disease 2019 (COVID-19) pandemic, the percentage of blood and respiratory cultures that tested positive for MDR organisms (MDRO) was higher than prepandemic levels.^[4]^ Studies conducted at selected hospitals in Albaha, Saudi Arabia, Brazil, and Italy have shown that patients with COVID-19 are more likely to be infected with MDRO than those without COVID-19.^[5–7]^ We conducted a search on the PUBMED database for studies pertaining to MDRO and hospital-acquired infections (HAIs) between the years 2018 and 2023. Together with the Centers for Disease Control and Preventions content on how germs are spread in healthcare facilities, we summarized the following 6 measures for review: improve hand hygiene compliance, environmental cleanliness, contact isolation, decolonization, cluster interventions, and early screening for pathogens.

## 2. Improve hand hygiene compliance

MDRO can be transmitted through contact with healthcare workers or surfaces contaminated with drug-resistant bacteria, as well as medical equipment in hospital environments.^[8]^ The World Health Organization (WHO) released the WHO Guidelines for the Implementation of Multimodal Hand Hygiene Improvement Strategies back in 2009.^[9]^ These guidelines put forward 5 moments for hand hygiene implementation: before healthcare workers come into contact with patients, before conducting clean/aseptic operations, after patient contact, after contact with the patient’s blood/bodily fluids, after contact with the patient’s environment, and strategies to improve hand hygiene. Implementing hand hygiene strategies such as infusing the infrastructure for hand hygiene, educating and training on hand hygiene, providing evaluation and feedback, setting up reminder tools in the workplace, and improving the concept of hand hygiene in the 5 areas is crucial. Hand hygiene improvement strategies can prevent 50% of avoidable infections that occur during healthcare while saving approximately 16 times the economic cost of implementation.^[10]^ It is widely agreed upon that hand hygiene implementation is necessary, and both Europe and the United States of America have incorporated it into norms regarding the prevention and control of nosocomial infections.^[11,12]^ However, implementing hand hygiene remains challenging. Results from the 2019 WHO Hand Hygiene Self-Assessment Framework global survey revealed moderate implementation of hand hygiene in 90 surveyed countries.^[13]^ Despite at least 3 studies demonstrating improved hand hygiene adherence among healthcare workers during the first wave of the COVID-19 pandemic,^[14–16]^ adherence gradually declined over time.^[17]^

One approach to enhancing compliance with hand hygiene among healthcare workers is through monitoring and feedback of its implementation. Presently, direct observation and the automated hand hygiene monitoring system (AHHMS) are commonly used monitoring techniques. In a tertiary hospital in Finland, an 8-year study^[18]^ was conducted to determine the effect of direct observation and feedback on the hand hygiene practices of healthcare workers. Forty-two observers were deployed across 13 wards to measure the duration of hand rubbing at each of the 5 hand hygiene moments, with over 10 monthly observations per ward and a total observation time of 4 to 6 hours per month per ward. The findings indicated a notably enhanced hand hygiene compliance of healthcare workers in all wards. Hand-washing compliance significantly improved in a US tertiary care hospital after a 1-month intervention. Trained observers stationed in the wards reminded medical staff to perform hand hygiene at the appropriate time. This intervention yielded positive results.^[19]^

An AHHMS was implemented at a hospital in Kentucky, USA. The medical staff wore wristbands equipped with motion sensors and signal receivers to record the time and position of their hand movements. The AHHMS determined if hand-washing practices met compliance standards and provided real-time feedback through wristband vibrations when necessary. After 16 weeks of use, the compliance rate for hand hygiene among medical staff improved.^[20]^ Iversen et al^[21]^ implemented an AHHMS in a 29-bed surgical ward. They placed sensors around the hand sanitizer dispenser and each bed, and Bluetooth sensors with distinct codes on the medical staff identification tags, so that the sensors could detect different code numbers when approached by the staff, either at the hand sanitizer dispenser or the bed. When a healthcare worker approaches a hand sanitizer dispenser or hospital bed, the sensor records the time and determines whether the healthcare worker has washed their hands before touching the patient based on the recorded time. The data collected by the sensors were provided to the healthcare workers every 2 weeks. After 14 months of use, there was a significant improvement in hand hygiene compliance among the healthcare workers. Another study found that implementing AHHMS was cost-effective.^[22]^

Other studies have improved hand hygiene compliance through role modeling, in addition to monitoring and feedback. Al-Maani et al^[23]^ conducted a study on role modeling in hand hygiene at 4 hospital settings. The study implemented measures such as appointing the hospital leader as a hand hygiene role model and displaying posters at entrances and corridors of wards. The hospital leader emphasized the importance of hand hygiene on a monthly basis. Additionally, each week, a healthcare worker was recommended as a hand hygiene role model, providing regular training and encouragement to other healthcare workers. Other measures were also taken to improve hand hygiene adherence. After a 3-month intervention, hand hygiene compliance showed improvement in all hospitals. Even after 15 months, compliance remained above 60%.

The aforementioned studies utilized different interventions that resulted in improved hand hygiene compliance among healthcare professionals. The use of AHHMS for hand hygiene has shown to be notably more effective and cost-efficient, thereby justifying its wider implementation.

## 3. Improved methods of cleaning and disinfecting the environment and facilities

Studies^[24]^ have indicated that methicillin-resistant *Staphylococcus aureus* (MRSA), vancomycin-resistant *Enterococci* (VRE), MDR *Pseudomonas aeruginosa*, and MDR *Acinetobacter baumannii* can persist on surfaces in hospital environments for extended periods. To prevent the transmission of MDROs, regular cleaning and disinfection of hospital facilities and equipment are crucial. Over the past 5 years, research on environmental and facility cleaning has predominantly focused on using ultraviolet (UV) light or hydrogen peroxide (HP) devices for disinfecting patient rooms after manual disinfection by cleaning staff, known as automated whole-room disinfection or whole-room disinfection (WRD).

UV is a general term for radiation in the electromagnetic spectrum, with wavelengths ranging from 10 to 300 nm, which can inhibit bacterial survival by affecting the activity of a variety of enzymes and destroying the structure of DNA. UV radiation is divided into 3 segments based on wavelength: UV A (UVA), UV B (UVB), and UV C (UVC). Findings from prior studies indicate that bacterial survival and activity are least affected by UVC.^[25]^ Yang et al^[26]^ utilized a UVC robot to disinfect hospital wards after patients with MRSA and VRE infections resided in them. They sampled and cultured the surfaces of the wards before and after disinfection and found a significant reduction in the number of MDRO colonies. Russo et al^[27]^ investigated the effectiveness of a specific UVC robot for disinfection and found fewer bacteria cultured on the ward surfaces after UVC disinfection compared to manual disinfection. Another UVC device, the pulsed-xenon-UV (PX-UV), is capable of irradiating a broad range of UVA, UVB, and UVC wavelengths. In Japan, Morikane et al^[28]^ employed a PX-UV device to disinfect the wards of discharged patients. The environmental surfaces of the disinfected wards were sampled and cultured. Compared to manual cleaning, the number of sampled and cultured colonies after disinfection by the PX-UV device was reduced by 59%. The disinfecting effectiveness of UVC is noteworthy; nevertheless, there are still limitations that need to be acknowledged. UVC can affect the function of normal cells in the human body, which is found in sunlight in the natural environment and is safer for humans than UVC. In a study by Livingston et al,^[29]^ it was observed that UVA irradiation for 4 hours led to a significant reduction in pathogenic microorganisms on medical equipment surfaces. This suggests that UVA devices may increasingly be used as safer UV disinfection devices in the future.

HP serves as an ideal nonpolluting disinfectant as it dismantles bacterial structures by producing oxygen radicals and breaking them down into water and oxygen. The primary techniques for WRD using HP currently encompass aerosolized HP (aHP), vaporized HP (VHP), and HP vapor.^[30]^ aHP devices typically disperse a 5% to 6% HP solution uniformly in the air as an aerosol to disinfect open environmental surfaces in a room. VHP devices emit hot vapors generated by a 30% to 35% HP solution through a high-velocity air stream in a confined space.^[30]^ Presently, a confusing narrative exists regarding aHP and VHP in the literature, where the method of operation is aHP but often mistakenly referred to as a VHP device. The aHP method, advocated by China’s Technical Code for Disinfection in Medical Institutions, recommends applying a 3% HP solution for 60 minutes of fogging disinfection with a dosage of 20 to 30 mL/m^3^. The VHP approach is not mentioned. Khandelwal et al^[31]^ investigated the effectiveness of a device utilizing a 7% HP solution for disinfection in intensive care wards. In comparison to manual cleaning, an aHP device reduced aerobic colony counts in the environment by 98% after a 42-minute operation. Rivera-Sánchez et al^[32]^ combined an 8% HP solution with 30 mg/L silver ions to disinfect stainless steel surfaces after MDRO contamination using a VHP device (which should actually be called an aHP device). Cobrado et al^[33]^ discovered that reducing the cycle time of the aHP system from the standard hour to 15 minutes did not result in variations in the growth inhibition of strains on different materials. Further research is required to determine the optimal concentration of the HP solution and the most effective duration for utilizing the aHP device.

Sanguinet and Edmiston^[34]^ conducted a study at 5 hospitals to assess the disinfecting efficacy of a device utilizing dry HP (DHP) technology. This technology converts moisture and oxygen in the air into anhydrous HP gas using plasma technology, eliminating the need for HP solutions. The device’s HP concentration was found to be safe for human exposure, remaining well below acceptable levels. During the use of DHP equipment, human presence was permitted, resulting in a 96.5% reduction in the average microbial load at the 5 hospitals on the first day of treatment. Another study conducted in an Australian hospital^[35]^ examined disinfection in an intensive care unit using a device similar to DHP, referred to as dilute HP. The study’s findings revealed no significant differences in microbial reduction between the use of DHP disinfection and no disinfection.

In addition to WRD, researchers are investigating the impact of self-cleaning metal-containing materials, such as copper and silver, on the spread of MDRO in hospital wards. Sifri et al^[36]^ conducted a study in which frequently touched surfaces like sinks and tables were replaced with copper oxide-infused materials, while copper-impregnated woven linen was used for items such as gowns and sheets. The intervention led to a 68% decrease in the incidence of MDRO infection among patients after 22 months. Meanwhile, Widmer et al^[37]^ introduced the use of silver-containing materials on high-touch surfaces like headboards, toilet seats, and dining tables. They observed pathogen reduction 2 months postintervention, and the effects remained sustained for 6 months. The study confirms that materials containing self-cleaning metals, such as copper and silver, effectively reduce MDRO. Enhancing high-touch surface materials in hospitals using these metals holds promise for diminishing MDRO infections in the future.

## 4. Contact precautions

Contact precautions are recommended for all patients with MDRO infections or those identified as MDRO-colonized, as advised by Centers for Disease Control and Prevention.^[38]^ These precautions primarily involve single-room occupancy, wearing isolation gowns and gloves before contact with patients or environments that could potentially be contaminated by patients, and donning isolation gowns and gloves before entering the patient’s room, removing them before exiting.

Iordanou et al^[39]^ implemented contact precautions for all hospitalized patients in intensive care units over 18 weeks, resulting in a decrease in the infection rate of MDR *P aeruginosa* after the intervention. On the other hand, a study by Bearman et al^[40]^ demonstrated that rigorous implementation of standard precautions in hospitals, even without contact precautions, did not lead to an increase in hospital infection rates. Granzotto et al^[41]^ conducted a questionnaire-based study comparing depression and psychological anxiety levels among patients receiving contact precautions and those not under such precautions. They discovered that patients under contact precautions exhibited higher levels of anxiety and depression.

In a separate study by Lee and Choi,^[42]^ patients under contact precautions were randomly divided into a control group and an intervention group. The intervention group received three 20-minute sessions of emotional support during the 1-week study period, involving expressing emotions, asking questions, and communicating, while the control group received no emotional support. The study revealed that participants in the intervention group had lower mean levels of anxiety and depression compared to those in the control group.

Current MDRO infection management guidelines in most regions now incorporate contact precautions,^[43,44]^ which have been associated with increased psychological distress for patients. It is suggested that regular emotional support may enhance patients’ psychological wellbeing. Further research is necessary to evaluate the overall effectiveness of contact precautions.

## 5. Decolonization

Decolonization involves treating patients colonized by specific MDROs, typically MRSA, to eliminate MDRO carriage.^[38]^ In a study by Huang et al,^[45]^ a decolonization protocol was implemented for discharged patients, which included chlorhexidine gargling, bathing, or showering with chlorhexidine, along with nasal mupirocin. Results after 6 months showed that discharged patients who underwent decolonization had a 30% reduced risk of MRSA infection compared to nondecolonized patients. However, an analysis conducted by Huang et al^[46]^ revealed no impact from this decolonization approach.

In a separate study, chlorhexidine bathing and nasal mupirocin were used to treat MRSA carriers outside the intensive care unit. The 21-month intervention period did not show a significant reduction in MRSA carriers following decolonization compared to conventional bathing. This was evidenced by the study’s results.

Further research is needed to explore the potential of oral administration of probiotics in reducing MDRO colonization among patients who have undergone antimicrobial drug use, as suggested by Wieërs et al.^[47]^ Additionally, while MDRO colonization in hospitalized healthcare workers has been studied, limited research exists on nonhospitalized healthcare workers. According to Rai et al,^[48]^ 9.4% of healthcare workers at a local hospital were found to be colonized by MRSA. These colonized workers underwent a 3-month 2% mupirocin nasal decolonization process, resulting in eradication of all MRSA infections. Screening healthcare workers for MDRO colonization is crucial to prevent MDRO transmission to patients.

## 6. Clusterization interventions based on evidence from the literature

Clusterization encompasses a series of evidence-based measures,^[49]^ combining effective strategies like hand hygiene practices and sterilization of healthcare environments. This approach refers to the amalgamation of these effective measures.

In 2021, Mody et al^[50]^ conducted a study employing a clustered intervention. Their approach included enhanced contact prophylaxis, chlorhexidine bathing, infection surveillance, education, feedback on environmental cleanliness, promotion of hand hygiene implementation, and healthcare worker education. After 2 years, the environment saw a 43% reduction in MDRO, although no significant change was observed in the prevalence of MDRO in patients.

Another study by Hall et al^[51]^ focused on deploying a cluster-based intervention to enhance environmental cleanliness in Australian hospitals. This intervention was crafted after identifying effective hospital cleaning interventions through a literature review. Experts from various fields were invited to prioritize these interventions, leading to the identification of 5 key components. These components comprised training sessions for cleaning staff to enhance hygiene practices, a standardized environmental cleaning sequence, focused cleaning of high-risk exposure areas, provision of appropriate cleaning products, assessment methods utilizing UV light or fluorescence for feedback, and improved communication channels. Researchers identified deficiencies in training, techniques, products, assessment, and communication using infection surveillance data and cleaning staff questionnaires.^[52]^ Following a 2-week training program addressing these gaps, the clusterization intervention was implemented across 11 hospitals for approximately 1 year. The study reported reduced rates of VRE infections, improved environmental cleaning, and increased staff knowledge levels.^[53,54]^ Additionally, the intervention was deemed 86% likely to be cost-effective.^[55]^ These findings suggest that such an approach offers a more efficient and cost-effective means of prevention and control.

## 7. Early screening for pathogens

Distinguishing between pathogenic species plays a pivotal role in directing the appropriate use of antimicrobial medicines, thereby aiding in controlling the incidence of hospital-acquired MDRO infections. In Switzerland, Darie et al^[56]^ conducted a randomized controlled trial involving patients suspected of having pneumonia. These patients were allocated to either a polymerase chain reaction (PCR) testing group or a routine microbiological testing group. Patients in the PCR group received advice on antimicrobial medication within 5 hours of sample delivery. Approximately 27 months later, the PCR group exhibited a 45.0% reduction in the duration of inappropriate antimicrobial use compared to the control group.

In a study by Yang et al,^[57]^ PCR was performed on rectal specimens from all patients admitted to the acute intensive care unit to screen for CRE, with results available within 1 hour after testing. After 3 years of active screening and infection control measures, a significant decrease in the rate of *Klebsiella pneumoniae* resistance was observed within the hospital.

Boere et al^[58]^ conducted a randomized controlled trial in the Netherlands involving patients suspected of respiratory tract infections. The intervention group, as determined by the physician, received immediate C-reactive protein monitoring, while the control group did not. After approximately 18 months, results showed that patients in the intervention group were 4.93 times more likely not to take antimicrobials at the time of initial diagnosis compared to the control group. These findings underscore the significance of early pathogen screening in patients suspected of bacterial infections in effectively controlling hospital-acquired MDRO infections.

## 8. Discussion

HAIs involving MDRO remain a significant concern. Recent research in the last 5 years has showcased promising outcomes in enhancing hand hygiene compliance through AHHMS. However, it’s imperative that physicians strictly adhere to the prescribed 5 moments of hand hygiene and follow standardized principles in healthcare settings. The practice of thoroughly cleaning and disinfecting healthcare environments plays a crucial role in curbing the transmission of MDRO. Encouraging the use of UV, HP technology, and self-cleaning techniques is also essential.

Contact isolation and decolonization are preventive measures based on pathogen colonization and transmission characteristics. Some studies conducted in the last 5 years have highlighted the effectiveness of these measures in controlling the spread of MDRO. Nevertheless, further rigorous randomized controlled trials are necessary to validate these findings. Early pathogen screening in patients suspected of infections can assist clinicians in determining appropriate antibiotic use, thereby reducing the emergence of antibiotic-resistant bacteria resulting from the misuse of antibiotics.

The prevention of HAIs with MDRO does not have a standardized intervention combination. Exploring clustered interventions might provide cost-effective combinations worth investigating further.

## Author contributions

**Data curation:** Binghui Ji.

**Writing – original draft:** Binghui Ji.

**Writing – review & editing:** Binghui Ji, Weijiang Ye.
